# Synchronization by the hand: the sight of gestures modulates low-frequency activity in brain responses to continuous speech

**DOI:** 10.3389/fnhum.2015.00527

**Published:** 2015-09-24

**Authors:** Emmanuel Biau, Salvador Soto-Faraco

**Affiliations:** ^1^Multisensory Research Group, Center for Brain and Cognition, Universitat Pompeu FabraBarcelona, Spain; ^2^Institució Catalana de Recerca i Estudis Avançats (ICREA)Barcelona, Spain

**Keywords:** audiovisual speech, gestures, beats, low-frequency oscillations, EEG

## Abstract

During social interactions, speakers often produce spontaneous gestures to accompany their speech. These coordinated body movements convey communicative intentions, and modulate how listeners perceive the message in a subtle, but important way. In the present perspective, we put the focus on the role that congruent non-verbal information from beat gestures may play in the neural responses to speech. Whilst delta-theta oscillatory brain responses reflect the time-frequency structure of the speech signal, we argue that beat gestures promote phase resetting at relevant word onsets. This mechanism may facilitate the anticipation of associated acoustic cues relevant for prosodic/syllabic-based segmentation in speech perception. We report recently published data supporting this hypothesis, and discuss the potential of beats (and gestures in general) for further studies investigating continuous AV speech processing through low-frequency oscillations.

Speakers spontaneously gesture to accompany their speech, and listeners definitely seem to take advantage of this source of complementary information from the visual modality (Goldin-Meadow, 1999). The aim of the present perspective is to bring attention to the relevance of this visual concomitant information when investigating continuous speech. Here, we argue that part of this explanation may have to do with the modulations that speaker’s gestures impose on low-frequency oscillatory activity related to speech segmentation in the listener’s brain. The speaker modulates the amplitude envelope of the utterance (i.e., the summed acoustic power across all frequency ranges for each time point of the signal) in a regular manner, providing quasi-rhythmic acoustic cues in at least two low-frequency ranges. First, speech syllables are produced rhythmically at frequency of 4–7 Hz, corresponding to a theta rate imposed by voicing after breath taking and jaw aperture (Peelle and Davis, 2012). Second, the speaker modulates pitch accents in her/his vocalization to convey particular speech acts (e.g., declarative or ironic), and emphasize relevant information to convey communicative intentions. These pitch peaks also occur with a quasi-rhythmic rate of 1–3 Hz corresponding to a delta frequency and constituting part of prosody (Munhall et al., 2004; Park et al., 2015). Recently, Electroencephalography (EEG) and Magnetoencephalography (MEG) studies investigated auditory speech segmentation mechanisms, taking advantage of time-frequency analyses to look at brain activities that are not time-locked to stimuli onsets, and measure the amount of activity in frequency bands of interest [typically missing in the classic Event-Related Potential (ERPs) averages]. These studies reported that spontaneous delta-theta activities in the auditory cortex reset their phase to organize in structured patterns, highly similar to the spectro-temporal architecture of the auditory speech envelope, reflecting entrainment mechanism (Ahissar et al., 2001; Luo and Poeppel, 2007; Abrams et al., 2008; Nourski et al., 2009; Giraud and Poeppel, 2012; Gross et al., 2013; Park et al., 2015; Zoefel and VanRullen, 2015). Then, delta-theta periodicity seems to constitute a fundamental window of compatibility between brain’s activity and speech segmentation (Ghitza and Greenberg, 2009; Peelle and Davis, 2012). Thus, when the natural delta-theta periodicity in the auditory signal is affected by time compression, speech comprehension worsens significantly. But more interestingly, the degradation of the delta-theta rhythms also decreases the spectro-temporal similarity between the speech envelope and the low-frequency activities in the auditory cortex (Ahissar et al., 2001). These important spectro-temporal features of the acoustic signal seem to be, therefore, important in determining brain responses to speech.

Yet, the acoustic signal is not the only communicative cue between speaker and listener. Coherent face and body movements often accompany verbalization. Before placing the focus on the speaker’s hand gestures, it is important to note that the relevance of non-verbal information has been first established regarding the speaker’s face (van Wassenhove et al., 2005). Corresponding lip movements have been long shown to facilitate comprehension in noisy conditions (Sumby and Pollack, 1954), or in contrast, affect speech processing when incongruent with utterance, e.g., the famous McGurk illusion (McGurk and MacDonald, 1976). More recently, visual speech information has been proposed to play a role in the extraction of the aforementioned rhythmic aspects of the speech signal (van Wassenhove et al., 2005). Due to the natural precedence of visual speech cues over their auditory counterparts in natural situations (i.e., the sight of articulation often precedes its auditory consequence; see Sánchez-García et al., 2011), it has been hypothesized that visual information conveys predictive information about the timing and contents of corresponding auditory information, facilitating its anticipation (van Wassenhove et al., 2005; Stekelenburg and Vroomen, 2007; Vroomen and Stekelenburg, 2010). For example, van Wassenhove et al. (2005) presented isolated consonant-vowel syllables in audio, visual or audiovisual modalities. They showed that the N1-P2 component in the auditory evoked responses time-locked to the phoneme onset were significantly reduced in amplitude and speeded up in time in the AV modality, compared to the responses to auditory syllables. In the time-frequency dimension, delta-theta entrainment has been proposed to underlie predictive coding mechanism based on the temporal correlation between audio-visual speech cues (Lakatos et al., 2008; Schroeder et al., 2008; Schroeder and Lakatos, 2009; Arnal and Giraud, 2012). Thus, Arnal and Giraud (2012) hypothesized that visual information provided by lip movements increases delta-theta phase resetting at relevant associated acoustic cue onsets (word onsets), reflecting predictive coding mechanisms that minimize the uncertainty about when regular event are likely to occur, and a better speech segmentation.

Along these lines, one could ask whether other speech-related visible body movements of the speaker may also bear predictive information and have an impact on low-frequency neural activity in the listeners’ brain. In continuous speech production, which movements may be correlated with delta-theta acoustic cues in the auditory signal? Head movements for example, were shown to be highly correlated with pitch peaks and facilitate comprehension of speech perception in noisy conditions (Munhall et al., 2004). Looking at public addressees, and in particular political discourses, we observed that speakers almost all the time accompany their speech with spontaneous hand gestures called “beats” (McNeill, 1992). Beats are simple and biphasic arm/hand movements that often bear no semantic content in their shape produced by speakers when they want to emphasize relevant information or develop an argument with successive related points. They belong to what could be considered as visual prosody, as they are temporally aligned with the prosodic structure of the verbal utterance, just like eyebrow, shoulders and head nods (McNeill, 1992; Krahmer and Swerts, 2007; Leonard and Cummins, 2011). Yasinnik (2004) showed that beats’ apexes (i.e., the maximum extension point of the arm before retraction, corresponding to the functional phase of the gesture) align quite precisely with pitch-accented syllables (peaks of the F0 fundamental frequency). In other words, the kinematics of beats match with spectro-temporal modulation of auditory speech envelope and are thought to modulate both the acoustic properties and the perceived saliency of the affiliated utterance (Munhall et al., 2004; Krahmer and Swerts, 2007). Albeit simple, beats have been found to modulate syntactic parsing (Holle et al., 2012; Guellaï et al., 2014), semantic processing (Wang and Chu, 2013) and encoding (So et al., 2012) during audiovisual speech perception. In a previous ERP study, we showed that the sight of beats modulate the ERPs produced by the corresponding spoken words at early phonological stages by reducing negativity of the waveform within the 200–300 ms time window (Biau and Soto-Faraco, 2013). Since the onsets of the beats systematically preceded affiliated words onsets by around 200 ms, we concluded that the order of perception and congruence between pitch accents and apexes attracted the focus of local attention on relevant acoustic cues in the signal (i.e., words onsets), possibly modulating speech processing from early stages.

Based on these previous studies and the stable spatio-temporal relationship between beats and auditory prosody, we argued that continuous speech segmentation should not be limited to the auditory modality, but also take into account visual congruent information both from lip movements and the rest of the body. Recently, Skipper (2014) proposed that listeners use the visual context provided by gestures as predictive information because of learned preceding timing with associated auditory information. Gestures may pre-activate words associated with their kinematics, to process inferences that are compared with following auditory information. In the present context, the idea behind was that if gestures provide robust prosodic information that listeners can use to anticipate associated speech segments, then beats may have an impact on the entrainment mechanisms capitalizing on rhythmic aspects of speech, discussed above (Arnal and Giraud, 2012; Giraud and Poeppel, 2012; Peelle and Davis, 2012). More precisely, we expected that if gestures provide a useful anticipatory signal for particular words in the sentence, this might reflect in phase synchronization of low frequency at relevant moments in the signal, coinciding with the acoustic onsets of the associated words (see Figure 1). This is exactly what we have tested in a recent EEG study, by presenting a naturally spoken, continuous AV speech in which the speaker spontaneously produced beats while addressing the audience (Biau et al., 2015). We recorded the EEG signal of participants during AV speech perception, and compared the phase-locking value (PLV) of low-frequency activity at the onset of words pronounced with or without a beat gesture (see Figure 1). The PLV analysis revealed strong phase synchronization in the theta 5–6 Hz range with a concomitant desynchronization in the alpha 8–10 Hz range, mainly at left fronto-temporal sites (see Figure 2). The gesture-induced synchronization in theta started to increase around 100 ms before the onset of the corresponding affiliate word, and was maintained for around 60 ms thereafter. Given that gestures initiated approximately 200 ± 100 ms before word onsets, we thought that this delay was enough for beat to effectively engage the oscillation-based temporal prediction of speech in preparation for the upcoming word onset (Arnal and Giraud, 2012). Crucially, when visual information was removed (that is, speech was presented in audio modality only), our results showed no difference in PLV or amplitude between words that had been pronounced with or without a beat gesture in the original discourse. Such pattern suggested that the effects observed in the AV modality could be attributed to the sight of gestures, and not just acoustic differences between gesture and no gesture words in the continuous speech. We interpreted these results within the following framework: beats are probably perceived as communicative rather than simple body movements disconnected from the message (McNeill, 1992; Hubbard et al., 2009). Through daily social experience, listeners learn to attribute linguistic relevance to beats because they gesture when they speak (McNeill, 1992; So et al., 2012), and seem to have an understanding of the sense of a beat at a precise moment. Consequently, listeners may rely on beats to anticipate associated speech segmentation that is reflected through an increase of low-frequency phase resetting at relevant onsets of accompanied words. In addition, it is possible that this prediction engages local attentional mechanisms, reflected by early ERP effects and the alpha activity reduction seen around word onsets with gesture. As far as we know, Biau et al. (2015) was the first study investigating the impact of spontaneous hand gestures on speech processing through low-frequency oscillatory activities in a close-to-natural approach. Further investigations are definitely needed to increase data and set new experimental procedures combining behavioral measures with EEG analyses.

**Figure 1:**
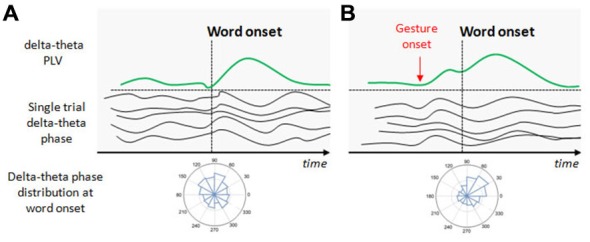
**Illustration of the potential effect of beat gestures on the delta-theta phase resetting. (A)** At the beginning of speech, neural populations in the auditory cortex spontaneously discharge at delta-theta rates but not at the same phase for a given time point (this is illustrated by the single trial delta-theta band phase before the word onset). At the first word onset, a phase distribution in the auditory sensors shows no preferred angle in the delta-theta band. In consequence, the delta-theta phase locking value (PLV) at the first word onset is weak. With progressive entrainment, delta-theta phase synchronizes, increasing PLV with a preferred angle at relevant syllable/word onsets. **(B)** Beat onsets systematically precede word onsets and potentially increase the delta-theta entrainment before the arriving word onset. When the relevant gesture onset occurs, delta-theta activity synchronizes with a preferred angle in the phase, increasing PLV before the associated word onset arrives to anticipate its processing.

**Figure 2:**
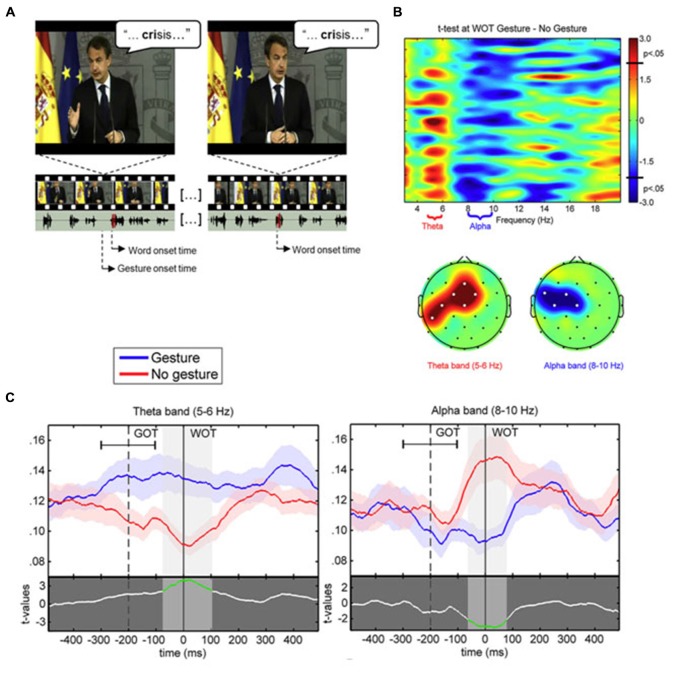
**(A)** Example of video-frames for the gesture (left) and no gesture (right) conditions associated to the same stimulus word “crisis”. The speaker is the former Spanish President Luis Rodríguez Zapatero, recorded at the palace of La Moncloa, and the video is freely available on the official website (Balance de la acción de Gobierno en 2010, 12–30–2010; http://www.lamoncloa.gob.es). Below, the oscillogram of corresponding audio track fragments (section corresponding to the target word shaded in red). The onsets of both the gesture and corresponding word (gesture condition) are marked. **(B)** (Tob) Representation of paired *t*-test values for the comparison between PLV at word onset in the gesture and no gesture conditions with frequency bands of interest labeled in the *x* axis. **(B)** (Bottom) Topographic representation of the significant clusters (significant electrodes marked with white dots) for the *t*-tests within the theta and alpha bands. **(C)** PLV time course in 5–6 Hz theta (left) and 8–10 Hz alpha (right) frequency bands at Cz electrode for the gesture (blue line) and no gesture (red line) conditions. The mean average ± standard deviation of gesture onset time (GOT) is represented respect to word onset time (WOT). The lower part of each plot displays the paired *t*-test values between gesture and no gesture conditions. The shaded bands indicate significant time intervals (highlighted in green in the *t*-test line).

A recent study by He et al. (2015) has investigated AV speech processing through low-frequency activity, albeit with a very different category of speech gestures. He et al. (2015) used intrinsically-meaningful gestures (IMG) conveying semantic content, such as when the speaker makes a “thumbs-up” gesture while uttering “the actor did a good job”. The authors investigated the oscillatory signature of gesture-speech integration by manipulating the relationship between gesture and auditory speech modalities: AV integration (IMG produced in the context of an understandable sentence in the listener’s native language), V (IMG produced in the context of a sentence in a foreign language incomprehensible for the listener) and A (an understandable sentence in the listener’s native language without gestures). The results of a conjunction analysis showed that the AV condition induced a significant centrally-distributed power decrease in the alpha band (7–13 Hz; from 700–1400 ms after the onset of the critical word associated with the gesture in the sentence), as compared to the V and A conditions that contained only semantic inputs from one modality (respectively: in the V condition only the gesture was understandable and in the A condition only the utterance was understandable). The authors concluded that the alpha power decrease reflected an oscillatory correlate of the meaningful gesture–speech integration process.

Investigations on the neural dynamics of hand gesture-speech integration during continuous AV speech perception have just begun but the results reported in both studies Biau et al. (2015) and He et al. (2015) already suggest two important conclusions for the present perspective. First, whereas auditory speech seems at first glance to attract all the listeners’ attention, hand gestures count as well, and may definitely be considered as visual linguistic information for online AV speech segmentation. If the delta-theta rhythmic aspects in the auditory signal can play the role of anchors for predictive coding during speech segmentation (Arnal and Giraud, 2012; Peelle and Davis, 2012; Park et al., 2015), then preceding visual gestural information, naturally present in face to face conversations, may convey very useful information for decoding the signal and thus, be taken into account. For instance, beats are not only exquisitely tuned to the prosodic aspects of the auditory spectro-temporal structure, but also engage language-related brain areas during continuous AV speech perception (Hubbard et al., 2009). This idea is in line with earlier arguments considering auditory speech and gestures as two sides of the same common language system (McNeill, 1992; Kelly et al., 2010; for some examples). Gestures may constitute a good candidate to investigate the multisensory integration between natural auditory speech and social postures. For example, Mitchel and Weiss (2014) showed that the simple temporal alignment between V and A information did not fully explain the AV benefit (i.e., multisensory integration) in a segmentation task of artificial speech. Indeed, segmentation was significantly better when visual information came from a speaker that was previously exposed to the words he had to pronounce during the stimuli recording (then, knowing the prosodic contours of words, i.e., boundaries), compared from a speaker that was unaware of word boundaries when recording. These results suggested that facial movements conveyed helpful visual prosodic contours if the speakers was aware of them. The same conclusion may apply to beat gestures as they synchronize with auditory prosody in communicative intent (and the speaker knows the prosodic contours of her/his own discourse). For example, it may be interesting to compare delta-theta activity patterns between gestures conveying the proper communicative prosody and simple synchronized hand movements without the adequate prosodic kinematics.

A second interim conclusion from the few current studies addressing the oscillatory correlates of gestures is that low-frequency brain activity appears to be a successful neural marker to investigate gesture-speech integration and continuous AV speech processing in general. Based on the results reported in these two pioneer studies, low frequency activity seemed sensitive to the type of gesture (intrinsically meaningful gestures in He et al., 2015; and beats in Biau et al., 2015). Both studies analyzed a contrast, comparing the low-frequency activity modulations between an AV gesture condition (i.e., words were accompanied with a gesture) and an AV no gesture condition (i.e., words were pronounced without gesture, but the speaker was visible). He et al. (2015) reported a decrease of alpha power (from 400–1400 ms) and a beta power decrease (from 200–1200ms) after the critical word onset, whilst Biau et al. (2015) reported a theta synchronization with a concomitant alpha desynchronization temporally centred on the affiliate word onset (note that the alpha activity modulation was found in both studies). Even if the experimental procedures and stimuli were not the same [in He et al. (2015) the speaker was still in the no gesture condition, whereas moving in Biau and Soto-Faraco (2013)], the distinct patterns of low-frequency modulations in the gesture-no gesture contrasts suggested that different kind of gestures may be associated to different aspects of the verbalization, modulating speech processing diversely. Indeed, IMGs describe a conventionally established meaning and can be understood silently whereas beats do not and need to be contextualized by speech to become functional. This might explain why the timing of modulations in He et al. (2015) was quite different respect to Biau et al. (2015). Then, oscillations may constitute an excellent tool for further investigations on neural correlate of AV speech perception and associated social cues with different communicative purposes (IMG vs. beats).

Speech is an intrinsically multisensory object of perception, as the act of speaking produces correlates to the ear and to the eye of the listener. The aim of the present short perspective was to bring attention to the fact that conversations engage a whole set of coordinated body movements. Furthermore, we argue that considering the oscillatory brain responses to natural speech may capture an important aspect of how the listeners’ perceptual system integrates back all the different aspects of the communicative production from the talker. Future studies may investigate more precisely how this integration occurs, and what is the role of synchronization and desynchronization patterns that we have tentatively interpreted here.

## Funding

This research was supported by the Spanish Ministry of Science and Innovation (PSI2013-42626-P), AGAUR Generalitat de Catalunya (2014SGR856) and the European Research Council (StG-2010 263145).

## Conflict of Interest Statement

The authors declare that the research was conducted in the absence of any commercial or financial relationships that could be construed as a potential conflict of interest.
